# Remimazolam tosilate compared with propofol for gastrointestinal endoscopy in elderly patients: a prospective, randomized and controlled study

**DOI:** 10.1186/s12871-022-01713-6

**Published:** 2022-06-10

**Authors:** Jian Guo, Yitao Qian, Xiaojin Zhang, Shuangjian Han, Qinye Shi, Jianhong Xu

**Affiliations:** 1 Department of Anaesthesiology, The Fourth Affiliated Hospital, Zhejiang University, School of Medicine, Yiwu, 322000 Zhejiang China; 2 Department of Obstetrics, The Fourth Affiliated Hospital, Zhejiang University, School of Medicine, Yiwu, 322000 Zhejiang China

**Keywords:** Remimazolam, Propofol, Gastrointestinal endoscopy

## Abstract

**Background:**

Remimazolam tosilate (HR7056, RT), a novel ultrashort-acting benzodiazepine, can be used for procedural sedation and general anaesthesia. However, few studies have focused on the sedative effect of RT during gastrointestinal endoscopy in elderly patients. The purpose of this study is to compare the sedative effect of RT and propofol for gastrointestinal endoscopy in elderly patients.

**Methods:**

A total of 82 patients aged ≥65 years with an American Society of Anaesthesiologists (ASA) grade I-II and a body mass index (BMI) of 18.0 to 30.0 kg/m^2^ who were scheduled for gastrointestinal endoscopy from Jan 2021 to Aug 2021 were selected and randomly divided into a RT group and a propofol group. Alfentanil 5 μg/kg was used for analgesia in both groups. The RT group was given remimazolam tosilate 0.15 mg/kg with supplemental doses of 0.05 mg/kg as need, while the propofol group was given propofol 1.5 mg/kg with supplemental doses of 0.5 mg/kg. The supplemental doses were determined by the modified observational alertness/sedation assessment (MOAA/S) score and the patients’ body movements. Sedative effects, such as the time to loss of consciousness (LOC) (MOAA/S score ≤ 1), successful sedation in one dose, number of supplemental doses after successful induction, and recovery time, were evaluated. Sedation-related side effects, such as injection pain, haemodynamic events and respiratory depression, were also noted. Postoperative nausea and vomiting (PONV), visual analogue scale (VAS) scores at rest, remedial analgesics, and dizziness or headache were recorded. In addition, patients’ satisfaction and physician’s satisfaction of the procedure were compared between the two groups.

**Results:**

Data from 77 patients were analysed. The success rate of sedation in both groups was 100%. The time to LOC (MOAA/S score ≤ 1) in the RT group was longer than that in the propofol group (20.7 ± 6.1s vs. 13.2 ± 5.2s, *P* < 0.001). There were fewer patients in the RT group reporting injection pain than that in the propofol group (0/39 vs. 5/38, *P* = 0.025). Haemodynamic events and respiratory depression in the RT group were less frequent than those in the propofol group ((6/39 vs. 17/38, *P* = 0.005), (2/39 vs. 9/38, *P* = 0.026), respectively). The number of supplemental doses after successful induction in the RT group was greater than that in the propofol group (4/9/11/13/1/1 vs. 8/4/18/6/2/0 requiring 0, 1, 2, 3, 4 or 5 supplemental doses, *P* = 0.014). The characteristics of the patients enrolled, postoperative parameters of the patients, and patients’ and physician’s satisfaction of the procedure were comparable in the two groups.

**Conclusions:**

Compared with propofol, RT can be safely and effectively used for gastrointestinal endoscopy sedation in elderly patients, and the incidence of sedation-related adverse reactions, especially haemodynamic events and respiratory depression, is lower. When RT is used, the number of supplemental doses after successful induction may increase slightly.

**Trial registration:**

Chictr.org.cn ChiCTR2000040498. Retrospectively registered (date of registration: December 1, 2020).

## Background

As the population ages, the incidences of benign and malignant gastrointestinal diseases increases. Endoscopic procedures are commonly performed in elderly patients and are very helpful in the screening and early diagnosis of gastrointestinal diseases [1]. With the requirement for comfortable medical treatment, painless endoscopic procedures have become mainstream. Elderly patients usually have a higher incidence of comorbid diseases and may be more susceptible to endoscopic interventions [2]. Due to their decreased physiologic reserve and associated diseases, elderly patients can have more severe complications than adult or young subjects [2, 3].

Propofol has excellent sedative properties in addition to a short half-life that allows rapid recovery, and it is widely used for sedation during painless endoscopic procedures [4]. However, propofol may cause adverse events to occur more frequently in elderly patients, such as hypoxemia, hypotension, bradycardia or tachycardia, arrhythmia, myocardial infarction, and even cardiac and/or respiratory arrest, which occur in proportion with the propofol dose [5–7]. Therefore, anaesthesiologists always seek sedatives with good sedative effects and relatively few adverse effects, which can be applied for endoscopic procedures in elderly patients.

Remimazolam, a member of the benzodiazepine family, is a novel ultrashort-acting gamma-aminobutyric acid a (GABA (A)) receptor agonist that may provide a new direction for sedation [8]. The pharmacokinetics and pharmacodynamics of remimazolam are related to its unique mechanism, the incorporation of a carboxylic ester moiety into the benzodiazepine core, which renders remimazolam susceptible to nonspecific tissue esterases and makes it rapidly metabolized into its pharmacologically inactive metabolite CNS 7054 [8]. Compared with midazolam, remimazolam produces a more rapid onset and a shorter duration of action. According to different stages of clinical trials and relevant studies, remimazolam can be used safely and effectively for procedural sedation (e.g., gastrointestinal endoscopy and bronchoscopy) and general anaesthesia [9–18]. Dai et al. [19] reported that remimazolam was a safe and effective sedative drug during induction, with fewer adverse effects for general anaesthesia in American Society of Anaesthesiologists (ASA) I or II patients. Remimazolam was also found to be safe and efficient for procedural sedation of high-risk ASA patients undergoing colonoscopy, which showed a safety profile that was comparable to that in low-risk ASA patients [18]. Schuttler et al. [20] found that the haemodynamics were relatively stable when remimazolam was used for continuous infusion in healthy male volunteers.

Remimazolam tosilate (HR7056, RT) developed by HengRui Medicine Co., Ltd., China has similar pharmacokinetics and pharmacodynamics to remimazolam [21–23]. Chen et al. [22] found that total treatment-emergent adverse events were decreased in the RT group compared to the propofol group; specifically, administration site pain, increased bilirubin, decreased respiratory rate and decreased SpO2 were less frequent, which showed that RT was safer than propofol, with fewer sedation-related adverse effects in patients undergoing colonoscopy. Therefore, we speculate that RT may be a better choice for sedation during painless endoscopic procedures in elderly patients. The main purpose of this research was to compare the sedative effect of RT and propofol for gastrointestinal endoscopy in elderly patients.

## Methods

### Study design

This study was a prospective, single-centre, randomized, controlled parallel-group clinical trial that compared the sedative effect of RT (HengRui Medicine, China) to propofol (Fresenius Kabi, AG) in elderly individuals undergoing gastrointestinal endoscopy at the Fourth Affiliated Hospital of Zhejiang University School of Medicine. This trial was approved by the Ethics Committee of the Fourth Affiliated Hospital of Zhejiang University School of Medicine (approval number: K2020067) and was registered in the Chictr.org.cn registration system on 01/12/2020 (ChiCTR2000040498). Informed written informed consent was obtained from all patients or their families following the CONSORT guidelines.

### Participants

A total of 82 patients who were scheduled for gastrointestinal endoscopy from January 1, 2021 to August 31, 2021 were selected. The patients were aged ≥65 years, with no restrictions regarding their sex. They were classified as ASA grade I-II and had a body mass index (BMI) of 18.0 to 30.0 kg/m^2^. Exclusion criteria included patients with contraindications for gastrointestinal endoscopy; severely difficult airways; apnoea syndrome; hypertension that was not satisfactorily controlled; abnormal liver or kidney function; history of opioid or other analgesic abuse; contraindications to benzodiazepines, opioids and propofol; and changes in endoscopic procedures due to gastrointestinal bleeding, gastrointestinal perforation, etc.

### Randomization

All eligible patients were randomly divided, using an Excel table, into one of two drug groups, a RT group and a propofol group, at a ratio of 1:1 by a medical worker not involved in the study. A single-blind design was employed because the colour, character and dosage forms of the two groups were different. Before the sedatives were administered, anaesthesiologists who were not involved in the study opened sealed envelopes to learn about the grouping situation. The study participants, postanaesthesia care unit (PACU) nurses, data statisticians, and outcome assessors were not aware of the test groupings.

### Anaesthesia

Patients who intended to undergo gastrointestinal endoscopy were instructed to prepare their gastrointestinal tract as needed, and the times of fasting and drinking were strictly controlled. Before the endoscopic procedures, the anaesthesiologists completed the preoperative evaluation and written informed consent forms.

After patients entered the room, ECG, blood pressure and oxygen saturation were monitored routinely, and the peripheral veins were opened. The baseline heart rate (HR) and mean arterial pressure (MAP) were defined as the average HR and MAP of the patients that were measured three times before the procedures. HR and oxygen saturation were monitored continuously during the perioperative period, and MAP was measured every 2.5 min (MAP was measured separately 1 min after induction). The entire anaesthesia process was performed by anaesthesiologists who were not involved in this study. In the RT group, total intravenous anaesthesia was induced with alfentanil 5 μg/kg and RT 0.15 mg/kg (slow intravenous injection, completed in 30 s), both according to the real body weight. In the propofol group, anaesthesia was induced with alfentanil 5 μg/kg and propofol 1.5 mg/kg (slow intravenous injection, completed in 30 s). During the entire gastrointestinal endoscopic procedure, sedation levels were assessed using the modified observational alertness/sedation assessment (MOAA/S) score by the anaesthesiologists every 30 s from 1 min to 3 min, then every 1 min, until three consecutive MOAA/S scores of 5 points [23]. When patients lost consciousness (MOAA/S score ≤ 1) [24], gastrointestinal endoscopy began [25]. If the MOAA/S scores were > 1 or physical movements occurred, up to a maximum of five supplemental doses administered as IV boluses (RT 0.05 mg/kg or propofol 0.5 mg/kg) were permitted after 1 min at the end of the initial dose [23, 25]. During the induction process, once the number of supplemental doses was more than 5, it was determined to be a failure of sedation, and sedative rescue medication (propofol) was administered. The occurrence of injection pain, the time to LOC (MOAA/S score ≤ 1) (defined as the time from the end of the sedative administration to loss of consciousness), the success rate of one-dose sedation, and the success rate of sedation were recorded.

During the procedure, once MOAA/S scores > 1 or physical movements occurred, supplemental RT 0.05 mg/kg or propofol 0.5 mg/kg doses were administered. After successful induction, the number of supplemental doses during the procedure was also recorded. Alfentanil 2 μg/kg was added for additional analgesia by the anaesthesiologists depending on the duration of the endoscopic procedures. Haemodynamic events and respiratory depression were recorded during the entire operation.

Patients were transferred to the PACU immediately after the procedure. The recovery time (the time from the last sedative administration to the awakening of the patients), the visual analogue scale (VAS) score at rest and remedial analgesics and sedative-related adverse reactions were recorded by the PACU nurses. Two hours after the procedure, patients were allowed to be discharged if they had postanaesthetic discharge scores ≥9.

Respiratory depression was defined as a respiratory rate < 8 times per min and/or blood oxygen saturation < 90%. Once respiratory depression occurred, mask pressurization was immediately initiated to assist ventilation, and tracheal intubation was performed if necessary. Haemodynamic events were defined as an intraoperative decrease in MAP and/or HR greater than 20% of the baseline value or systolic blood pressure ≤ 80 mmHg. When haemodynamic events occurred, fluid therapy was administered immediately (rapid intravenous infusion of normal saline 200 ml). If the effect of fluid resuscitation was not good, vasoactive medication was selected (ephedrine 6 mg or phenylephrine 40 μg IV, depending on the HR).

The patients’ satisfaction was assessed according to whether they were aware of the procedure, whether they experienced dizziness, nausea or vomiting after the procedure or other factors. The physician’s satisfaction was evaluated according to the procedure conditions, whether the procedure was interrupted, etc. Full-satisfaction scores for both assessments totalled 10 points, with 0-3 points defined as unsatisfactory, 4-7 points as relatively satisfactory, and 8-10 points as satisfactory.

### Outcomes

The primary outcomes were the differences in the success rate of sedation, the time to LOC (MOAA/S score ≤ 1) and the recovery time between the RT group and the propofol group. Secondary outcomes included differences between the two groups in terms of injection pain, successful sedation in one dose, haemodynamics at 1 min after induction, haemodynamic events, respiratory depression, number of supplemental doses after successful induction, VAS score at rest, use of remedial analgesics, sedatives related adverse reactions, patients’ and the physician’s satisfaction of the procedure.

### Sample size estimation and statistical methods

In a preliminary experiment of six cases per group, we found that the success rate of sedation in both groups was 100%, so we chose the time to LOC (MOAA/S score ≤ 1) as the main reference factor. The time to LOC (mean ± standard deviation) was 20.6 ± 2.6 s in the RT group and 10.6 ± 2.0 s in the propofol group. Therefore, the effect size of the two groups was 0.90. The required minimum sample size for each group was 34 (calculated by a t test, a 2-sided test, a level of significance of 0.05, and a power of 0.95). The total sample size was 82 to allow for an approximately 20% dropout rate.

Statistical testing was conducted with SPSS 22.0. Categorical variables are presented as absolute numbers. Normally distributed data are expressed as the mean ± standard deviation ($\overline{x}$ ± SD). Normally distributed data were compared among multiple groups using single-factor analysis of variance (ANOVA). Nonnormally distributed data were compared among multiple groups using the Kolmogorov–Smirnov test. Grade count data were assessed by the χ2 test. A difference in *P* < 0.05 was considered statistically significant.

## Results

### Participants

**Fig. 1:**
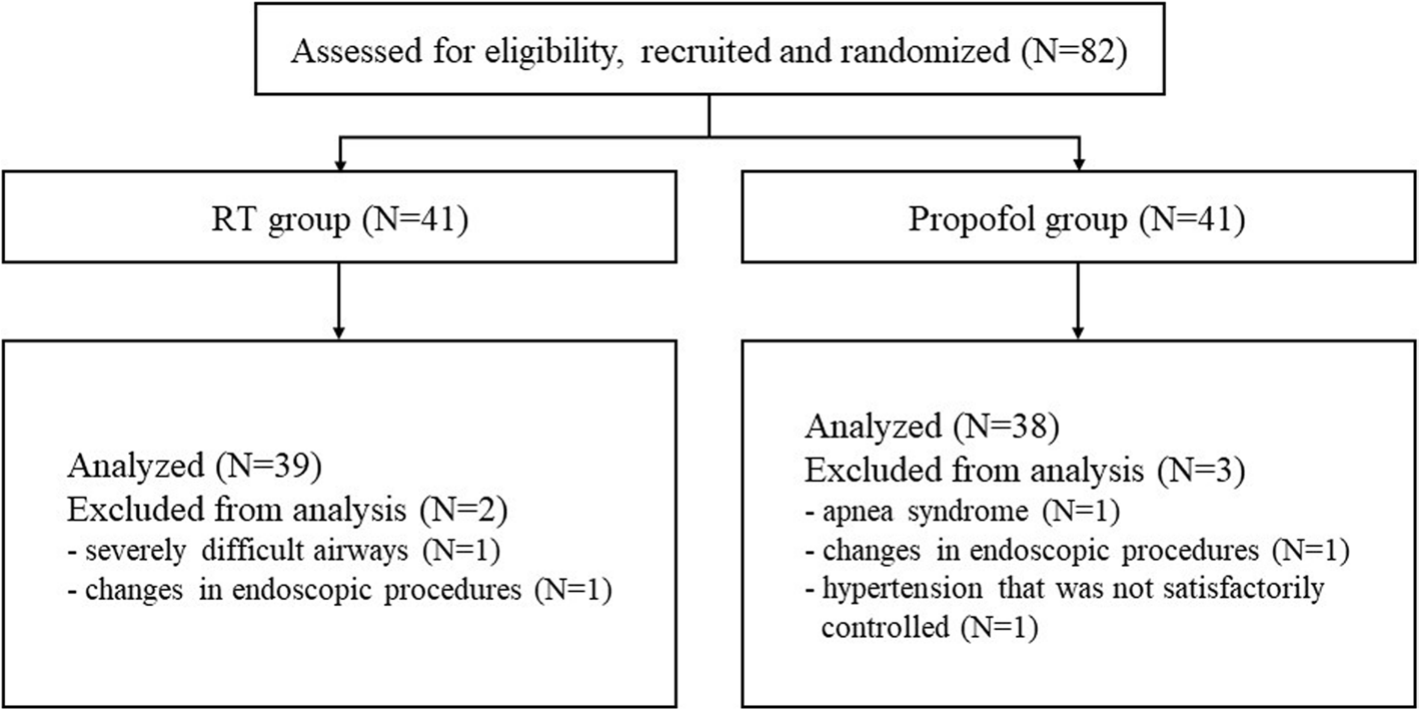
Patient recruitment, randomization and withdrawal. Five of the 82 patients withdrew for various reasons, and 39 patients from the RT group and 38 patients from the propofol group were eventually included in the final analysis

A total of 82 patients who were scheduled for gastrointestinal endoscopy from Jan 2021 to Aug 2021 were selected. Two patients in the RT group and three patients in the propofol group withdrew for various reasons, and data from 77 patients were ultimately analysed, as shown in the CONSORT flow diagram (see Fig. 1).

### Basic information

The characteristics of the enrolled patients are summarized in Table 1. No significant differences in age, sex, height, weight, BMI, ASA classification or medical comorbidities (including hypertension, diabetes mellitus, coronary heart disease, chronic obstructive pulmonary disease and history of surgery) were noted between the RT group and the propofol group (*P* > 0.05) (see Table 1).

**Table 1:** Characteristics of the patients enrolled (*N* = 77)

	RT group (*N* = 39)	Propofol group (*N* = 38)	*P value*
Age (years)	70.4 ± 3.9	69.1 ± 4.0	0.185
Sex (male/female)	25/14	22/16	0.373
Height (cm)	164.3 ± 7.5	163.0 ± 8.1	0.459
Weight (kg)	62.3 ± 9.5	61.3 ± 11.1	0.678
BMI (kg/m^2^)	23.0 ± 3.0	23.0 ± 3.4	0.958
ASA classification (grade I/II)	7/32	7/31	0.595
Medical comorbidities			
Hypertension (N.)	16	16	0.554
Diabetes mellitus (N.)	4	5	0.483
CHD (N.)	5	4	0.483
COPD (N.)	6	4	0.385
History of surgery (N.)	21	17	0.284

Data are expressed as the frequencies or means ± SDs, as appropriate

*BMI* Body mass index, *ASA* American Society of Anaesthesiologists, *CHD* Coronary heart disease, *COPD* Chronic obstructive pulmonary disease

### Information on gastroenteroscopy and anaesthesia

Information concerning gastroenteroscopy and anaesthesia is listed in Table 2. No significant differences in the duration of gastroenteroscopy, procedure category, infusion volume, or alfentanil consumption were noted between the two groups (*P* > 0.05) (see Table 2).

**Table 2:** Information on gastroenteroscopy and anaesthesia (*N* = 77)

	RT group (*N* = 39)	Propofol group (*N* = 38)	*P value*
Duration of gastroenteroscopy (min)	17.3 ± 8.2	17.5 ± 6.8	0.911
Procedure category (N.)			
(Gastroscopy/Colonoscopy/Gastrointestinal endoscopy)	5/11/23	7/7/24	0.540
Infusion volume (ml)	225.8 ± 75.2	245.0 ± 71.0	0.256
Alfentanil consumption (μg)	329.9 ± 61.9	324.5 ± 85.4	0.751

Data are expressed as the frequencies or means ± SDs, as appropriate

### Sedative effects

Information on sedative effects between the two groups is shown in Table 3. The success rate of sedation in both groups was 100%. No significant differences in terms of successful sedation in one dose and recovery time were noted between the two groups (*P* > 0.05) (see Table 3). The time to LOC (MOAA/S score ≤ 1) was longer in the RT group than in the propofol group (20.7 ± 6.1 vs. 13.2 ± 5.2, *P* < 0.001) (see Table 3). The number of supplemental doses after successful induction seemed to be greater during procedures in the RT group than during those in the propofol group (4/9/11/13/1/1 vs. 8/4/18/6/2/0, *P* = 0.014) (see Table 3).

**Table 3:** Comparison of sedative effects between the two groups (*N* = 77)

	RT group (*N* = 39)	Propofol group (*N* = 38)	*P value*
Time to LOC (MOAA/S score ≤ 1)(s)	20.7 ± 6.1*	13.2 ± 5.2	**< 0.001**
Successful sedation in one dose (N.)	35	36	0.350
Number of supplemental doses after successful induction (0/1/2/3/4/5) ^a^	4/9/11/13/1/1*	8/4/18/6/2/0	**0.014**
Recovery time (min)	12.3 ± 3.2	12.9 ± 4.2	0.495

Data are expressed as the frequencies or means ± SDs, as appropriate

LOC = loss of consciousness

^a^ Patients with different numbers of supplemental doses during gastroenteroscopy were counted in both groups

* *P*<0.05 compared to the Propofol group

### Sedation-related side effects

All sedation-related side effects of the two groups are listed in Table 4. No significant differences in terms of MAP or HR pre-operation, PONV, dizziness or headache were noted between the two groups (*P* > 0.05) (see Table 4). Injection pain was reduced in the RT group compared with the propofol group (0/39 vs. 5/38, *P* = 0.025) (see Table 4). Compared with those in the propofol group, higher MAP and HR values were observed 1 min after induction in the RT group ((72.1 ± 4.9 vs. 68.0 ± 4.1, *P* < 0.001), (58.6 ± 3.8 vs. 56.3 ± 3.9, *P* = 0.026), respectively). Both the incidence of haemodynamic events and respiratory depression of patients were lower in the RT group than in the propofol group ((6/39 vs. 17/38, *P* = 0.005), (2/39 vs. 9/38, *P* = 0.026), respectively)). There was no postoperative agitation, postoperative delirium or skin pruritus in the two groups.

**Table 4:** Comparison of sedation-related side effects between the two groups (*N* = 77)

	RT group (*N* = 39)	Propofol group (*N* = 38)	*P value*
Injection pain (N.)	0*	5	**0.025**
Haemodynamics			
Preoperation			
Baseline MAP (mmHg)	79.0 ± 4.4	79.5 ± 4.2	0.652
Baseline HR (bpm)	65.5 ± 4.2	67.0 ± 4.1	0.118
One minute after induction			
MAP (mmHg)	72.1 ± 4.9*	68.0 ± 4.1	**< 0.001**
HR (bpm)	58.6 ± 3.8*	56.3 ± 3.9	**0.026**
Haemodynamic events (N.)	6*	17	**0.005**
Respiratory depression (N.)	2*	9	**0.026**
PONV (N.)	10	8	0.419
Dizziness/headache (N.)	11	14	0.286

Data are expressed as the frequencies or means ± SDs, as appropriate

*MAP* Mean arterial pressure, *HR* Heart rate, *PONV* Postoperative nausea and vomiting

* *P*<0.05 compared to the Propofol group

### Pain assessment, remedial analgesics and patients’ satisfaction and physician’s satisfaction of the procedure

The VAS score at rest, remedial analgesics and patients’ satisfaction and physician’s satisfaction of the procedure are listed in Table 5. No significant differences in terms of VAS score at rest, remedial analgesics or patients’ satisfaction and physician’s satisfaction of the procedure were noted between the two groups (*P* > 0.05) (see Table 2).

**Table 5:** Comparison of postoperative pain assessment, remedial analgesics and patients’ satisfaction and physician’s satisfaction of the procedure between the two groups (*N* = 77)

	RT group (*N* = 39)	Propofol group (*N* = 38)	*P value*
VAS score at rest	1.2 ± 1.0	1.3 ± 1.1	0.583
Remedial analgesics (N.)	1	2	0.490
Patients’ satisfaction	8.2 ± 0.6	8.0 ± 0.6	0.323
Physician’s satisfaction	8.0 ± 0.9	8.1 ± 0.8	0.445

Data are expressed as the frequencies or means ± SDs, as appropriate

*VAS* Visual Analogue Scale

## Discussion

The purpose of this study was to compare the sedative effect of RT and propofol used for gastrointestinal endoscopy in elderly patients. In our study, we found that RT had a slower onset of sedation in elderly individuals, but the incidence of related side effects was lower, especially the incidence of haemodynamic events and respiratory depression. There was no significant difference in the recovery time between the two groups. In addition, the number of supplemental doses after successful induction may have increased slightly during the procedure in the RT group.

Remimazolam is a new type of water-soluble ultrashort-acting anaesthetic sedative. It is hydrolysed and metabolized by nonspecific tissue esterases. By binding to the GABA(A) receptor, the opening frequency and permeability of the chloride ion channel of the nerve cell membrane are increased, allowing chloride ions to enter the cell under the condition of a concentration gradient, leading to an increase in the intracellular membrane potential, hyperpolarization and decreased excitability, which inhibits the electrical activity of neurons and produces a sedative effect [26]. Due to its special methyl propionate side chain mechanism, remimazolam showed a high clearance (1.15 ± 0.12 l/min, mean ± SD), a small steady-state volume of distribution (35.4 ± 4.2 l) and a short terminal half-life (70 ± 10 min) [20]. As a new type of benzodiazepine, the sedative effect of remimazolam can be quickly reversed by its antagonist flumazenil [10]. Based on reported clinical trials, remimazolam has demonstrated its effectiveness and safety with promising properties, including a rapid onset, a short duration of action, a predictable and consistent recovery profile, and metabolism almost unaffected by liver or renal function, with no or minimal cardiorespiratory depression and availability with a reversal drug [17]. Moreover, remimazolam does not prolong cardiac repolarization [27]. Compared to remimazolam, RT has a similar pharmacokinetics and pharmacodynamics [21–23]. All these advantages guarantee the application of RT for sedation during endoscopic procedures in elderly individuals. Referring to the drug instructions and relevant literature, as well as considering the particularity of the elderly population, a lower dose of RT (initial dose of 0.15 mg/kg, supplemental doses of 0.05 mg/kg as needed) was selected, and its safety and effectiveness were confirmed by our preliminary experiments [19].

Chen SH et al. [23] confirmed that the sedation success rate of the RT group was 97.35%, while our investigation revealed that the rate of sedation success of RT was 100% in preliminary experiments and performed trials, which may be related to two reasons. First, the object of our study was the elderly population over 65 years old. Compared with young adults, elderly adults may have less demand for sedatives, and the success rate of sedation will be higher. Second, compared with the previous trial using a fixed dose of RT (initial dose of 5.0 mg, with supplemental doses of 2.5 mg as needed), we used individualized medication based on weight (initial dose of 0.15 mg/kg, supplemental doses of 0.05 mg/kg as needed), which was more in line with the standard of individualized medication.

Propofol is an intravenous hypnotic that is widely used for endoscopic procedural sedation. Phillips et al. [28] found that the median (interquartile range) propofol dose in the group aged > 65 years was 1.8 (1.4-2.2) mg/kg, that is, above the recommended dose, in comparison to 2.2 (1.9-2.5) mg/kg in younger patients. Therefore, in this study, a reduced dose of propofol (initial dose of 1.5 mg/kg, supplemental doses of 0.5 mg/kg as needed) was selected, and its sedative effect were also confirmed by our preliminary experiments. The ranges of published noncompartmental PK parameters for adults after propofol infusion are a half-life of fast distribution of 1.33–4.6 min, a half-life of slow distribution of 27–69.3 min, a half-life of elimination of 116–834 min, a mean residence time of 102–174 min, and a total blood clearance of 1.78–2.28 L/min, which may explain why the propofol group has a faster onset time [29]. Similar to that in other studies, the time to LOC (MOAA/S score ≤ 1) was longer in the RT group than in the propofol group. The onset time of sedation in this study was relatively fast, which may be related to the dosage and method of drug administration. In our study, the success rate of one-dose sedation induced by RT was significantly higher than that in other trials, mainly because we chose the induction dose according to a body weight of 0.15 mg/kg (significantly higher than the fixed dose of 5 mg in some cases). Since RT was mainly metabolized by nonspecific tissue esterases and had a shorter half-life, we found that the number of supplemental doses after successful induction seemed to be greater during procedures in the RT group than during those in the propofol group.

Propofol is almost an ideal IV anaesthetic agent, but the overall risk of pain from propofol injection alone is approximately 60% [30]. Pain on propofol injection (POPI) is the seventh most important problem in the current practise of clinical anaesthesia and may be related to skin, mucous membrane and vascular involvement [31]. Unlike propofol, RT is not a phenol and therefore poses less irritation to tissues. Similar to the results of another study, no injection pain was found in the RT group [23]. The main adverse reaction of propofol is dose-related cardiopulmonary depression. In the preliminary experiment, we found that patients in the RT group had higher EEG bispectrum monitoring values among the successfully sedated patients, suggesting that the depth of sedation in the propofol group was deeper than that in the RT group. The deeper the sedation depth, the more obvious cardiopulmonary fluctuations were often predicted. On the other hand, Win et al. [32] found that propofol enhances the dominance of parasympathetic activity, which was associated with decreased HR and arterial blood pressure (BP). Midazolam enhances the dominance of sympathetic activity, which is associated with increased HR and decreased BP. RT, similar to midazolam, may also have an effect on sympathetic activity, leading to lower cardiopulmonary depression.

In a previous study, RT (5.75 min) showed a faster recovery from sedation than propofol (6.71 min) [23]. However, we found that the recovery time of the two groups of patients was significantly prolonged, and we did not find that RT yielded a faster recovery from sedation than propofol. The main reason for the above phenomenon may be related to the elderly population in this study. The drug metabolism time of elderly patients is relatively prolonged, the recovery time increases accordingly, and lower doses can be considered [16].

Our study has some limitations. First, the sedation target (MOAA/S score ≤ 1) was relatively deep, and the dose of propofol selected might have been slightly larger for elderly patients, which may have led to excessive sedation and an increase in the incidence of sedative-related adverse events. Second, the node of this study was 2 hours after the procedure, and there was no follow-up for the occurrence of related adverse events in the 3 days after the procedure, which might have led to differences in the results. Third, this investigation mainly focused on the clinical control of pharmacodynamics without relevant pharmacokinetic inspections. Fourth, we did not use EEG bispectrum to monitor and compare the depth of sedation throughout the whole process, which may lead to differences in the depth of sedation affecting the results of the study. Fifth, this was a single-centre, randomized, prospective study, and further large-sample, multicentre studies are still needed to confirm this conclusion.

## Conclusions

Compared with propofol, RT can be safely and effectively used for gastrointestinal endoscopy sedation in elderly patients, and the incidence of sedation-related adverse reactions, especially haemodynamic events and respiratory depression, is lower. When RT is used, the number of supplemental doses after successful induction may increase slightly.

## Data Availability

The datasets generated and/or analysed during the current study are not publicly available due to institutional restrictions but are available from the corresponding author on reasonable request. The email address of the corresponding author is 1197058@zju.edu.cn.
